# The effect of iron supplement on children with euthyroid goiter: a randomized placebo-controlled clinical trial 

**Published:** 2014-07-20

**Authors:** M Ordooei, M Akbarzadeh, R Soleimanizad, F Shamsi, R Masoumi Dehshiri

**Affiliations:** 1 Department of Pediatric Hematology oncology and Genetic Research Center, Shahid Sadoughi University of Medical Sciences and Health Services, Yazd, Iran; 2 Shahid Sadoughi University of Medical Sciences and Health Services, Yazd, Iran; 3 MSc in biostatistics, Department of biostatistics and epidemiology, Faculty of health, Shahid Sadoughi University of Medical Sciences and Health Services, Yazd, Iran; 4 Health Policy Research Center, Shahid Sadoughi University of Medical Sciences and Health Services, Yazd, Iran

**Keywords:** Iron, goiter, treatment

## Abstract

**Background:**

Endemic goiter is present in most parts of Iran. Iron deficiency adversely affects the physiology of thyroid. The initial steps of thyroid hormone synthesis are catalyzed by thyroperoxidases and are dependent on iron. In many developing countries, children are at high risk of both goiter and iron deficiency anemia. In addition, iron deficiency may alter central nervous system control of thyroid metabolism; andalso Iron-deficiency anemia decreases plasma concentrations of thyroxine and triiodothyronine.

**Material and method:**

We conducted a randomized, double-blind,controlled trial in 2-12-y-old children with euthyroid goiter and without iron-deficiency anemia.

The children weredivided into two groups: The Patients who were under treatment with ferrous-sulphat and controls .active treatment consisted of iron supplementation was administered orally with a dose of 2 mg/kg day. The duration of active treatment was 12 weeks.

**Results:**

In this study 40 children (female: 22, male: 18) were divided into two groups randomly. (20 patients in each group).There were no significant differences at baselines between groups with regard to gender, age and weigh.

At the end of the study, the reduction of more than one grade of goiter was significant between iron-treated and placebo groups. In treatment group, 16 patients (80%) had decreased grade of goiter, while in the control group, 3 patients (15%) had grade reduction(P-value<0.001).

**Conclusion:**

At the end of the study, decrease of more than one grade of goiter had significant differences between iron-treated and control groups.

## Introduction

Children may have goiters that are diffuse or nodular.the goiters may be associated with normal, decreased, or increased thyroid hormone production. The causes of goiter in children and adults are similar, but their relative frequency varies substantially. In the United States, for example, most children with a goiter have chronic autoimmune thyroiditis, whereas among adults, nontoxic nodular goiters predominate (1).

Euthyroid goiter is a condition with normal serum thyroid-stimulating hormone in the setting of normal total or free thyroxin (T4) concentration in serum (2).

Iron deficiency adversely affects the physiology of thyroid and supplementation of iron may improve the efficacy of oral iodized oil in goitrous children with iron-deficiency anemia (3).

Iron status also affects thyroid metabolism and iodine deficiency disorders (IDD).The initial steps of thyroid hormone synthesis are catalyzed by thyroperoxidases and are depended on iron. In addition, iron deficiency may alter central nervous system control of thyroid metabolism (4) and modify nuclear triiodo thyronine binding (5). 

Iron-deficiency anemia decreases plasma concentrations of thyroxine and triiodo thyronine, while reducing the peripheral conversion of thyroxine to triiodo thyronine, and increases circulating concentrations of thyrotropin (4,6,7).

 In goitrous children, the therapeutic response to orally given iodized oil is lower in children with iron deficiency anemia compare to iron-sufficient children (8). In addition, in an open, uncontrolled trial, iron treatment of goitrous children with iron deficiency anemia improved their response to orally given iodized oil (9). 

Therefore, the aim of this study was to determine whether iron treatment would decrease the grade of goiter in children with euthyroid goiter without anemia and iron deficiency or not.

## Materials and Methods

**In order to compare the effectiveness of iron **supplementation or placebos for euthyroid goiter, we conducted a randomized, double-blind, active-controlled trial on 40 patients with euthyroid goiter without iron-deficiency anemia.

Random sampling was performed to complete the sample size, Taking sample size at 95% and 80% test power, and considering 60% reduction in goiter size in case group and 20% in the control group. The minimum 40% difference between two groups and the minimum sample size required per group was 20 samples.

n = (Z1-α/2 + Z1-β)2 × [P1 (1-P1) + P2 (1-P2)] /(P1- P2)2

Newly diagnosed individuals with euthyroid goiter were selected to study at the outpatient Endocrinology and Metabolism clinic in Shahid Sadoughi University of Medical Sciences, Yazd, Iran from January to May 2014.

A total of 40 patients with euthyroid goiter (thyroid-stimulating hormone_4.5-10 mU/L and normal fT4 and fT3) were visited in the clinic. None of the patients had iron-deficiency anemia (hemoglobin_12 g/dL and ferritin_25 _g/L for women; hemoglobin_13.5 g/dL and ferritin_30 _g/L for men).

Inclusion criteria were as below:

1-Children age 7 to 9 years

2-The child who suffers from goiter

3-Without iron-deficiency anemia

Exclusion criteria:

1-Have a history of allergy to drugs

2- Mental retardation

3- Patients taking iron or iron supplements

All selected patients have been examined clinically by pediatric endocrinologist and his assistant for proper selection to the trial study. Assigned measurements were performed on subjects sitting up right with the neck extended.

Thyroid size was determined by inspection and palpation. History-taking and physical examination with attention to the cause of goiter and measurement of thyroid-stimulating hormone (TSH) for the evaluation of thyroid function were performed.

Classification or grading of goiter:

1a: A goiter that is palpable but not visible when the neck is in extended position.

1b:Can be seen at a perspective in the extended position

2: When the examiner sitting next to him, a goiter is clearly visible

3: Can be seen from a distance.

In cases that differences were observed in the grade measurements by an endocrinologist and assistant pediatric oncology, goiter grade measured by the endocrinologist was accepted.

All patients were evaluated by an endocrinologist and a hematologist before and after therapy. Medical history and physical examination were performed in the initial diagnostic evaluation of euthyroid.

Blood was collected by venipuncture for the measurement of hemoglobin, serum ferritin, T4, TSH andsent to Central Laboratory (referral laboratory).

Serum ferritin was measured with ELISAReader (Awareness model, USA).

Anti TPO, TSH and T4 were measured according to Radio Imionency method with gamma Gantro- Cell Counter.

In children aged 2 - 12 years old, hemoglobin<11.5 g/dl and ferritin<12ng/mL were considered as anemia and iron deficiency,respectively(11).

After the interview and parental consent, children divided in to two groups. The patients group Patients who were under treatment with ferrous- sulphat and control group who received placebo. 

Studies were eligible for inclusion if they met the following criteria. Active treatment consisted of iron supplementation was administered orally with a dose of 2 mg/kg /day. The duration of active treatment was 12 weeks.

Patients were instructed to have a normal diet and advised to avoid consumption of mineral or vitamin supplements and an excssive amount of tea and bran foods.

All the patients were evaluated twice (before and after therapy)during the study period(during the three months of treatment).

All patients were able to contact the clinic by telephone during the study period as needed. Of note, 100% of the patients (N_40) completed the study per protocol.

The effectiveness of the drug was defined as a reduction of more than one grade in the grade of goiter.

**Statistical Analysis**

Forty patients equally randomized between two groups and sexes with in each group were used to evaluate the objectives of the study. Normally distributed data are reported as mean standard deviation, and count data are reported by N (%). Paired t test was used to compare before and after measurements in each treatment group for each of the primary and secondary end points.

Analysis of variance model was used to compare changes from baseline in hemoglobin, serum ferritin, and thyroid stimulating hormone across treatment groups and baseline characteristics (age, gender). A p- value=.05was considered statistically significant. Data processing and statistical analysis were performed by using SPSS 15.

## Results

Forty children (female: 22, male: 18) were divided into two groups randomly (20 patients in each group).

No treatment-related adverse events were reported in any of the treatment and control groups. All patients were asymptomatic and had no iron deficiency and have euthyroid goiter. 

Characteristics of the iron-treated and placebo groups at baseline were compared and there were no significant differences at baseline between groups with regard to gender, age and weigh.

There were no statistically significant differences in the grading of goiter before the study in treatment and control groups, but after three months of treatment with ferrous sulphate there were significant differences between two groups (Table I).

At the end of the study reduction of more than one grade of goiter was significant between iron-treated and placebo groups (Table II).

Reduction by more than one grade in the grade of goiter in each group was not significantly different between men and women (p=0/052)

There were no significant differences between men and women in treatment groups in grade Ib(p=1)

Iron treatment significantly reductin in grade II in women (Table III). Frequency of side effect in treatment group with ferrous sulphate was 35%.

**Table I:** Frequency distribution of goiter grades in both groups before and after treatment

**Group** ** grade**	**Before treatment with iron**		**after treatment with iron**	
	Treatment group	control group	Treatment group	control group
**Ia**	0(0%)	0(0%)	11(55%)	2(10%)
**Ib **	13(65%)	14(70%)	8(40%)	14(70%)
**II **	7(35%)	6(30%)	1(5%)	4(20%)
**III **	0(0%)	0(0%)	0(0%)	0(0%)
**p-value**	1		0/008	

**Table II:** Reduction of more than one grade of goiter

**Group ** **decrease** ** of more than one grade of goiter**	**Control group **	**Treatment group**	**p-value **
Yes	3 (15%)	16 (80%)	**> ** **0.001**

**Table III:** Comparison the frequency distribution of sex

**Goiter grade** **sex**	**Garden Ia**	**Garde Ib**	**garde II**	**Total**
Women	1(100%)	0	0	**1(100%)**
men	0	5(83%)	1(16/7%)	**6(100%)**

## Discussion

Diseases of thyroid, namely goiter, hypothyroidism and hyperthyroidism, constitute the most common endocrine abnormality in recent years and are common in children (10). However, goiter is a severe 

public-health problem according to the WHO/UNICEF/ICCIDD-recommended criteria (11) and is asymptomatic in most cases (12), This study evaluated the role of iron in the treatment of patient euthyroid goiter patients whitoutanemia. 

There are limited reports on interaction between the goiter rate and the iron status (13).

In this study, iron maintenance dose of 2mg/kg was effective in reducing the grade of goiter in children with 9-7 years old without iron deficiency.

In the Philippines, there was no difference in goiter rate between anemic and non-anemic subjects (14). In two studies in Iran and Ethiopia, no correlation was found between the iron status and the goiter rate or thyroid hormone levels (15, 16). In another trial, addition of encapsulated iron to iodized salt improved the efficacy of iodine in goitrous children with a high prevalence of anemia (17).

A cross-sectional study performed on school children in Isfahan in 2005 showed that the goitrous children had a higher iron-deficiency rate than the non- goitrous ones (18). A similar finding was reported from a recent study in Iran where iron deficiency was associated with an increased rate of goiter (19). The mechanism by which the iron status influences thyroid and iodine metabolism is unclear (20).In this study, we conducted a new study to evaluate the possible reduction the goiter grade with treatment of iron supplement.

In addition, iron deficiency may alter the control of thyroid physiology in the central nervous system and modify nuclear T3 binding (21, 22). In the present study, there was no significant difference in the mean TSH and T4 levels between the iron-deficient and the iron-sufficient subjects that was similar to our study. 

Treatment with iron supplement in euthyroid goiter patient, improves thyroid function.

Hess SY et al in randomized, double-blind, placebo-controlled trial in 5-14-y-old children in Côte d'Ivoire found that the hemoglobin and iron status at 20 wk were significantly better after iron treatment compared to placebo (P < 0.05). At 20 wk, the mean reduction in thyroid size in the iron-treated group was nearly twice thanin the placebo group (x +/- SD percentage change in thyroid volume from baseline: -22.8 +/- 10.7% compared with -12.7 +/- 10.1%; P < 0.01). At 20 wk, goiter prevalence was 43% in the iron-treated group compared with 62% in the placebo group (P < 0.02)(23).

In descriptive cross sectional research on school children in mashhad in 2007-2009 showed the prevalence of iron deficiency anemia in young girls are moderate (24).

Our study was different from that we evaluated effects of iron inpatient with normal ferritin in all grades of goiter, Separation and interfere with sex.

Sex had no difference in reducing the grade of goiter, but ingrade separation,reducing the grade of goiter was effective inFemales in Grade II which can indicate the role of sex in high grade and that maybe girls are at greater risk of iron deficiency or goiter.

Further research should be performed in girls with high grade goiter.

Reduction in size was not significantly differentin in various grades, but it was higher in samples ingrade I. At baseline there was no grade III, Maybe because that in this age, the highest prevalence of euthyroid goiter is Grade Ib or represented that the most effect of iron in the primary grades.

Because in higher grades, symptomatic children are considered as patient to investigate the effectiveness of iron need a longer treatment period.

Only35% of patients who had received iron had been complicated, that were occasional abdominal pain and other side effects that were not observed.

In addition, none of the side effects led to stop the treatment.

This result show that is useful effects of iron in children with euthyroid goiter. 

## Conclusion

At the end of the study, decrease of more than one grade of goiter had significant differences between iron-treated and control groups.iron deficiency is associated wiht goiter in children and these children may benefit from iron supplementation.
